# Comparative Value of the Different Preparations of Mercury and Iodine, and the Best Modes of Administering Them

**Published:** 1844-11

**Authors:** Edward Octavius Hocken

**Affiliations:** Physician to the Blenheim-street Infirmary


*Comparative value of the different preparations of Mercury and Iodine, and the best modes of administering them.* By Edward Octavius Hocken, M.D., &c., Physician to the Blenheim-street Infirmary.—Mercury is employed locally and generally, either to produce a local effect simply, or, by its admission into the sestem, to bring the whole constitution under its influence. The mercurial influence is induced in the system by the introduction of mercurial preparations into the stomach, or by fumigation, or by inunction. In the first method we employ the chloride, bichloride, iodide, *pil. hydrarg.*, *&c.*, *&c.*

*Chloride.*—Calomel is chiefly useful when we wish to produce a speedy and powerful action on the constitution as in venereal iritis or orchitis, but is less adapted to the ordinary symptoms. On the ontinent it is extensively employed in tubercles of the labia, with or without ulceration, in various forms of creeping ulcers, and also in ulcerations of the throat and nasal fossæ. Desruelles says, that he cannot too much recommend this preparation, which united to opium and an antiphlogistic regimen, may produce the most beneficial results. Ricord employs the following pills in the treatment of enlarged testicle, which remains after inflammation of that organ.

Hyd. Chor. ℈i. Pulv. Conii, Sapon. Hisp. aa ℈ij. M. ft. pil. xxiv.

*Bichloride.*—M. Dupuytren ordered this remedy in small doses, one-sixth of a grain three times a day, in constitutional syphilis, and on the Continent it still continues to be extensively used for this purpose. In some chronic cases of of syphilitic skin disease, I have seen it used with advantage; but as a general remedy in secondary syphilis it requires more care, is more dangerous, and altogether is a less eligible medicine than the blue pill.

*Pilula Hydrargyri.*—This medicine is the form most used and relied on in England, and as it is one of the mildest, safest, most certain, and most manageable preparations of mercury, it justly deserves the preference given to it. In doses of five grains two or three times a day, it is applicable to nearly all those conditions which we have shown to be benefitted by mercury.

*Proto-ioduret.*—MM. Cullerier, Biett, Ricord and others employ this remedy in many forms of constitutional syphilis, especially where secondary and tertiary symptoms are combined, and in primary sores in strumous habits. Cullerier says, that it is chiefly in constitutional syphilis that the proto-ioduret of mercury is administered with success. Its effects are principally evident in secondary ulcerations of the mucous membrane, cutaneous tubercles, exostoses, and chronic affections of the joints, where the other preparations of mercury have had little effect. It should always be guarded by opium, and given in half grain doses twice or thrice a day. The deuto-ioduret is more stimulating, and consequently its dose is smaller. Either of these may be employed in friction upon tumours and idolent buboes, after the removal of all acute inflammatory symptoms.

The cyanuret and deuto-phosphate of mercury are occasionally employed. The former is said to be preferable to the bichloride, being less apt to disagree, and less readily decomposed. It is an useful external application in some skin affections, allaying the violent itching and irritation of what M. Alibert terms *herpes squamosus.*

*Inunction.*—Inunction by the mercurial ointment was formerly employed to mercurialize the system more frequently than at the present day. In this way the mineral is less apt to disagree with the system, especially the alimentary canal, although, when used alone, it is less speedy in its effects. In buboes, I imagine that Hunter was correct in his opinion concerning the advantages of making mercury pass through the affected absorbents. The *ung. hydrarg.* is used in the quantity of half a drachm to a drachm night and morning, to be well rubbed in, before a fire, on the more delicate portions of the skin. Cullerier prefers using mercury by friction in primary sores; he orders from a quarter of a drachm to a drachm and a half of mecurial ointment at each friction, leaving an interval between them of one, two, or three days, with the view of not irritating either the sore or the constitution, by bringing the latter suddenly under the influence of the remedy. Ricord frequently orders them to the axillæ, and they are employed in this manner by Cullerier, in certain forms of ulcerations of the mouth and fauces. He narrates two cases cured by mercurial frictions in this situation, which had resisted its employment in other parts.

*Fumigation.*—Fumigation of the whole surface of the body is, at present, rarely used as a method of affecting the system, but the apparatus formerly employed is still to be found in some of our hospitals. It is very speedy in its action. The remedy is, however, employed locally, and with gerat advantage, in some affections of the throat and nasal fossæ, directed to the part by a suitable apparatus, and more generally in some obstinate diseases of the skin. For patients who have not strength to rub in mercury, and whose bowels will not bear the use of internal remedies, it has been esteemed highly advantageous.

*Iodine.*—M. Cullerier thinks that the effects of the iodide of potassium are less prompt than those of mercury, and that, on this account, more should be given, if the stomach will bear it. He employs grain doses of iodine with from two to four of the iodide of potassium in an ounce of water, given at intervals during the day; but he does not increase the iodine beyond two grains in the day, or the iodide beyond ten. I fully believe that the iodide is much more beneficial without the pure iodine, which disorders the stomach without benefitting the complaint. Mr. Stone, formerly apothecary to St. Thomas's Hospital, told Dr. Williams that he was called to prescribe for ten patients taking the compound of iodine and iodide of potassium for one that was taking the last medicine only.

Dr. Wallace found by experience that the iodide of potassium was the only form of the remedy which agreed, that pure iodine was a very powerful irritant, very frequently occasioning severe symptoms, whilst the iodide of potassium was quite harmless. Pure iodine, moreover, is converted into hydriodic and in the stomach. He has seen many cases in which the tincture of iodine, both simple and ioduretted, failed to produce any favorable influence, because the irritation excited in the stomach prevented its employment in such doses as were sufficient to act on the disease, and in these very cases the action of the iodide of potassium was subsequently most beneficial. In other cases, where pure iodine was employed, although the disease was cured, still it was at the expense of an injured stomach, and great emaciation. On the contrary, he asserts that he has never seen unpleaant effects result from the iodide of potassium, except from mismanagement.

Ricord employs the iodide of potassium in gradually increasing doses, commencing with ten grains dissolved in three ounces of distilled water, and given at intervals during the day, in any suitable vehicle. According to effects so must the dose be either increased or diminished;—when the remedy agrees, which it always does, if the stomach be healthy, the dose should be increased ten grains every two or three days, till it is carried to one and one and a half drachms, or even more, in the coutse of the day. The iodide of potassium, in full doses, when it agrees, occasions a sensation of warmth in the stomach, improves the appetite, accelerates digestion, so that more grow quite fat, and quickens the pulse. A constant effect is an increased diuresis.

When pure iodine is used, or the iodide given in excessive quantities, or from idiosyncracy of constitution, unpleasant symptons may arise. Sometimes those are slight, and resemble a common catarrh; at others, ringing in the ears, and pain in the head, or the skin may suffer from a slight pustular eruption; occasionally it disorders the bowels, or produces pain or uneasiness in the stomach, having some resemblance to pleurodynia, but seated more deeply, and an acrid dryness of the throat. Mr. Mayo says that we may sometimes correct these symptoms by adding a few drops of laudanum to each dose, and by administering aperient medicine. Authors assert that some patients experience idiotic intoxication, characterized by a slight uncertainty in the voluntary movements, some *subsultus tendinum*, heaviness in the head, a species of intellectual idleness. and sometimes delirium. Soreness of the gums and ptyalism are also said to occur occasionally. Mr. Mayo has heard of effects resembling mercurial erythismus. Should any of these symptoms occur in a severe degree, the dose must be diminished, or even abandoned altogether for a few days, and its exhibition re-commenced in smaller doses.

Dr. Wallace found the urine to be the best test of the effects of the iodide of potassium on the system. by testing it with starch, &c. In some of his patients he remarked a great increase of perspiration—sometimes constipation, salivation, roughness of the throat, and heartburn; he found that quinine controlled the state of the throat and stomach. Delicate females, he says, sometimes lose the power of sleeping so much as is natural—a state of wakefulness often accompanied by peculiar feelings of the head, which is relieved by a purgative and interruption of the medicine. Emaciation, great gastric irritation, wasting of the mammæ and testes, &c., only occur from the free use of iodine. In two patients who had drachm doses of the iodide of potassium administered by mistake for one day, there occurred in both sickness, soreness of the throat, colicky pains, vomiting and purging to a slight degree, frequent pulse, and exhaustion, quickly disappearing. Several patients, while under the full action of the iodide, were attacked with an acute pain in the anterior and lower part of the left side, precisely in the centre of the superficeis formed by the false ribs, accompanied by some cough, difficulty of breathing and fever. In all, the affection went off without much trouble. The medicine was omitted and subsequently resumed without inconvenience. In a private patient it produced severe indigestion, a rapid and quivering pulse, headache, and a peculiar condition of the eyes—the pupils were dilated, and both eyes in a state of incessant motion. He was soon after seized with symptoms of paralysis on one side of his body, preceded by muscular tremblings, which remained for three weeks, but eventually passed off.

Ricord states that the good effects of the iodide of potassium have been constant in his practice, but not produced with equal rapidity, in this respect differing from Mr. Mayo, who says that no medicine, where it does good, produces amendment so rapidly; therefore the propriety of continuing it is never doubtful. As far as I have observed myself, iodide of potassium never gives rise to any serious symptom, provided that it be unmixed with pure iodine, and be administered in moderate doses. Apoplectic and paralytic symptoms sometimes come on during the existence of tertiary symptoms, and these are then attributed to the mercury or the iodine which the patient may be using at the time, but it is hardly fair that the whole blame should fall on the remedy. For an adult it is sufficient to commence with five grains of the iodide of potassium three times a day, and increase it gradually to seven or eight. Dr. Williams, while he admits that some constitutions are affected even by one or two grains, thinks that the average dose should be eight grains three times a day; for, says he, a smaller dose can hardly be recommended, for the patient's sufferings are so intense as to require immediate relief, and consequently we ought to begin with as large a dose as his stomach will probably bear. This reasoning is not altogether conclusive, for if the dose be sufficient to excite or endanger unpleasant symptoms, we shall have to stop its use altogether for some time, and then to resort to smaller doses, which, if used at first, would most probably have removed the complaint without any distress or delay. Dr. Williams remarks, that when mercury has been previously and successfully used, the quantity of the iodide necessary for the cure of the patient is often much greater than where none has been exhibited.

*Review of the comparative value of Mercury and Iodine in the treatment of Syphilis.*—If we take a review of what has been written, we can readily determine the comparative value of mercury and iodine in the treatment of syphilis—that mercury and iodine form the two main remedies on which the best and most unprejudiced treatment of the various symptoms and stages of syphilis mainly hinges, although neither of them should be regarded as a specific, nor can either of them, to be used well and successfully, be exhibited empirically;—that mercury and iodine, when guided by observation, reason, and experience, and combined with such treatment and medicines as the profession would employ were they to lay aside all notions of something specific requiring a blind and specific use of some remedial agent, they stand alone, and infinitely superior to all other medicines which the materia medica can furnish; that a modified use of mercury is adapted to nearly all the forms, but especially the indurated, of primary syphilis; that in constitutional syphilis a modified use of mercury is almost a *sine qua non* in the great majority of secondary symptoms, but is either hurtful or useless in the tertiary; that iodine is inert in almost all the symptoms of primary syphilis, with the exception of some forms of phagedena, attended with great debility and derangement of the health; that in constitutional syphilis it is a less valuable remedy to the majority of secondary symptoms than mercury, with the exception of some severe cases of pustular eruption, phagedenic throat, rupia, and secondary ulcerations of bad character, all of them marked by a cachhetic and debilitated constitution; while in tertiary symptoms iodine is far more valuable than mercury, and its effects more certain and decided than in any other set of symptoms;—that mercury and iodine are most advantageously combined in cases presenting both secondary and tertiary symptoms;—that many forms of mercury, having local or constitutional actions, are applicable to the various symptoms of syphilis, but that the mildest constitutional effect, capable of overcoming the disease, is always to be preferred;—that the only form of iodine safely applicable to the treatment of syphilis, is the iodide of potassium, which should never be carried beyond moderate doses; hence, however valuable the iodide of potassium may be in some forms of syphilis, it cannot be substituted with advantage for mercury in the great majority.—Braithwaite from *Edin. Med. Surg. Journal.*

